# Characterization of a Time-Resolved Diffuse Optical Spectroscopy Prototype Using Low-Cost, Compact Single Photon Avalanche Detectors for Tissue Optics Applications

**DOI:** 10.3390/s18113680

**Published:** 2018-10-29

**Authors:** Mrwan Alayed, Darek P. Palubiak, M. Jamal Deen

**Affiliations:** 1 School of Biomedical Engineering, McMaster University, Hamilton, ON L8S 4L8, Canada; alayedms@mcmaster.ca; 2 National Nanotechnology Center, King Abdul Aziz City for Science and Technology (KACST), Riyadh 11442, Saudi Arabia; 3 Department of Electrical and Computer Engineering, McMaster University, Hamilton, ON L8S 4L8, Canada; palubid@mcmaster.ca

**Keywords:** diffuse optical spectroscopy, time-resolved spectroscopy, tissue optics, single-photon avalanche diode, silicon photodetectors, time-correlated single-photon counting

## Abstract

Time-resolved diffuse optical spectroscopy (TR-DOS) is an increasingly used method to determine the optical properties of diffusive media, particularly for medical applications including functional brain, breast and muscle measurements. For medical imaging applications, important features of new generation TR-DOS systems are low-cost, small size and efficient inverse modeling. To address the issues of low-cost, compact size and high integration capabilities, we have developed free-running (FR) single-photon avalanche diodes (SPADs) using 130 nm silicon complementary metal-oxide-semiconductor (CMOS) technology and used it in a TR-DOS prototype. This prototype was validated using assessments from two known protocols for evaluating TR-DOS systems for tissue optics applications. Following the basic instrumental performance protocol, our prototype had sub-nanosecond total instrument response function and low differential non-linearity of a few percent. Also, using light with optical power lower than the maximum permissible exposure for human skin, this prototype can acquire raw data in reflectance geometry for phantoms with optical properties similar to human tissues. Following the MEDPHOT protocol, the absolute values of the optical properties for several homogeneous phantoms were retrieved with good accuracy and linearity using a best-fitting model based on the Levenberg-Marquardt method. Overall, the results of this study show that our silicon CMOS-based SPAD detectors can be used to build a multichannel TR-DOS prototype. Also, real-time functional monitoring of human tissue such as muscles, breasts and newborn heads will be possible by integrating this detector with a time-to-digital converter (TDC).

## 1. Introduction

Diffuse optical spectroscopy (DOS), also called near-infrared spectroscopy (NIRS), is non-invasive and non-destructive technology to determine the optical properties (OP) of turbid objects such as tissues in which scattering dominates absorption [1,2,3]. DOS exploits the low absorption and high scattering of tissues in the optical window (also called biological window) at red and near-infrared wavelengths (650–900 nm) [4,5]. In this range of wavelengths, light can diffuse in the tissue and penetrate up to a few cm [5]. Light propagation in turbid media is well described by the radiative transfer equation (RTE) and its simplified version, the diffusion equation (DE) [6]. Hence, re-emitted photons can be detected either in transmittance geometry (source and detectors not on the same side), or in reflectance geometry (source and detector on the same side of the object) for thin and thick objects [3,7]. The detected signal using DOS can be analyzed using an inverse problem solver to retrieve the OP, particularly the reduced scattering coefficient (*μ_s_*’) and the absorption coefficient (*μ_a_*) [4,8]. Therefore, the OP of a homogeneous object or the distribution of OP for a heterogeneous object can be recovered [9,10]. DOS measurements are made using three main approaches: continuous-wave (CW), frequency-domain (FD), and time-resolved (TR) [5,11].

Continuous-wave diffuse optical spectroscopy (CW-DOS) has limitations in retrieving the absolute values of *μ_s_*’ and *μ_a_* because this approach depends on one quantity (changes of light intensity) which only allows for estimating the change of *μ_s_*’ and *μ_a_* values [3,4]. Both FD-DOS and TR-DOS can estimate the absolute values of *μ_s_*’ and *μ_a_* with good accuracy [12]. However, TR-DOS has depth selectivity because it discriminates between early and late photons in the histograms of the raw data [13]. This is an important feature in functional brain imaging for retrieving the absolute values of *μ_s_*’ and *μ_a_* in multilayered objects such as a human head [4,13,14]. However, current TR-DOS requires complex, expensive equipment and costly computation to analyze the raw data [2,15]. These requirements limit the use of TR-DOS systems for potential applications such as nondestructive optical characterization of pharmaceuticals, food, wood, and for clinical applications such as muscle monitoring, functional brain imaging and optical mammography [1,2]. Therefore, significant efforts are being made to simplify the complexity of TR-DOS systems to develop and utilize affordable instruments and to analyze the experimental data using efficient computational methods [1,2,16,17] As a result, it is expected that compact, low cost and portable multichannel TR-DOS systems will be available in the near future [1,2,18].

**Figure 1:**
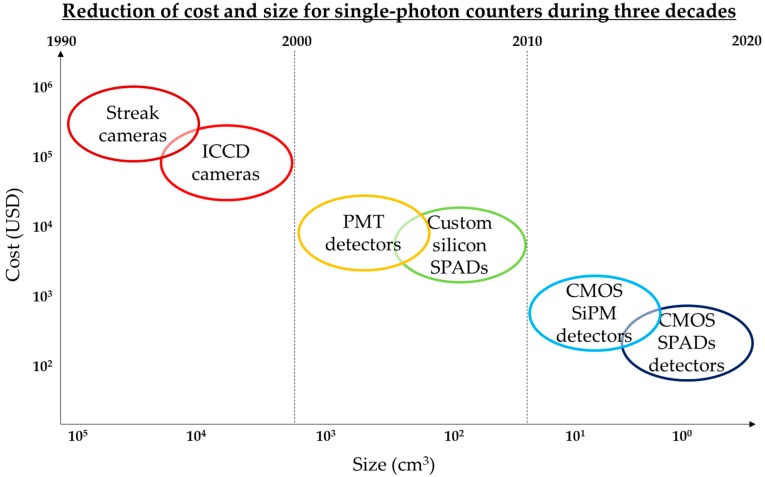
Evolution of single-photon counters in according to their size and cost.

The main advances in reducing the cost and the size for instruments used for single-photon timing applications such as TR-DOS are achieved due to the recent developments in silicon photonics technologies [1,19]. Using technologies such as the complementary metal-oxide-semiconductor (CMOS) silicon technology simplify the implementation and the fabrication for the solid-state detectors [1,2,20]. These detectors, mainly SPADs and SiPMs, are much smaller and cheaper than sophisticated traditional photon timing equipment such as Streak cameras, ICCD cameras, and PMTs detectors as shown in Figure 1 [1,2].

Therefore, several CMOS SPAD detectors have been reported recently for single-photon timing applications, and some of them have been validated for TR-DOS applications [21,22]. Although CMOS SPAD detectors have lower performance versus SPAD detectors based on custom silicon technologies, they have the potential to be used in building low cost and compact photon timing systems for tissue optics applications using several available options of standard CMOS technologies [1,20]. Therefore, smaller dimensions CMOS technologies such as 130 nm, 90 nm, 65 nm can be utilized to miniaturize the size of SPAD arrays, and to integrate timing electronics without significant degradation of the fill factor of the detector in comparison with larger dimensions CMOS technologies (800 nm and 350 nm) [23]. For instance, the fill factor of the SPAD detectors are in the range of 1%, 9%, and 25% for 800 nm 350 nm, and 130 nm CMOS technologies, respectively [24]. Towards this target, we have developed a compact free-running (FR) single-photon avalanche diodes (SPADs) in a standard, low-cost, digital 130-nm CMOS technology that builds on our previous works on SPADs [25,26,27].

In this work, we built a TR-DOS prototype using our FR-CMOS SPAD detectors to investigate the importance of each performance feature for the FR-CMOS SPADs in TR-DOS measurements since some desired characteristics of the CMOS SPAD detectors need to be studied, as explained in our recent review [1]. The main desired characteristics of the CMOS SPAD detectors are low levels of noise (dark count rate and afterpulsing), short timing jitter, large fill-factor and good photon detection efficiency. When good performance features are achieved for one pixel SPAD detector, it is possible to replicate the pixel to build an array of SPADs [20]. Also, we report on a comprehensive evaluation of this TR-DOS prototype, and we demonstrate its capability when used for tissue optics applications. The evaluation of this prototype was achieved in two different levels that included the instrument performance and the quantification of the OP of realistic phantoms. Our prototype has a short total instrument response function (*IRF_Total_*) and low differential non-linearity (DNL). The accuracy assessments showed low average errors that were less than 10% for retrieving *μ_s_*’ and *μ_a_* for several homogeneous phantoms. Also, this prototype demonstrates good linearity and can be used to estimate differences in the OP values among realistic phantoms.

## 2. TR-DOS Prototype

The main components of this TR-DOS prototype can be classified into three parts. The first part is the light illumination subsystem-pulsed laser source and associated electronics. The second part is the photon counting and timing subsystem. These two parts represent the TR-DOS setup which generates raw data called the distribution of time of flight (DToF) histograms for the re-emitted photons from a phantom. The third part is the data analysis software which preprocesses the DToF histograms and recovers the OP of the phantoms using a best-fitting model. Figure 1 illustrates the main components of the TR-DOS prototype.

### 2.1. Light Sources

**Figure 2:**
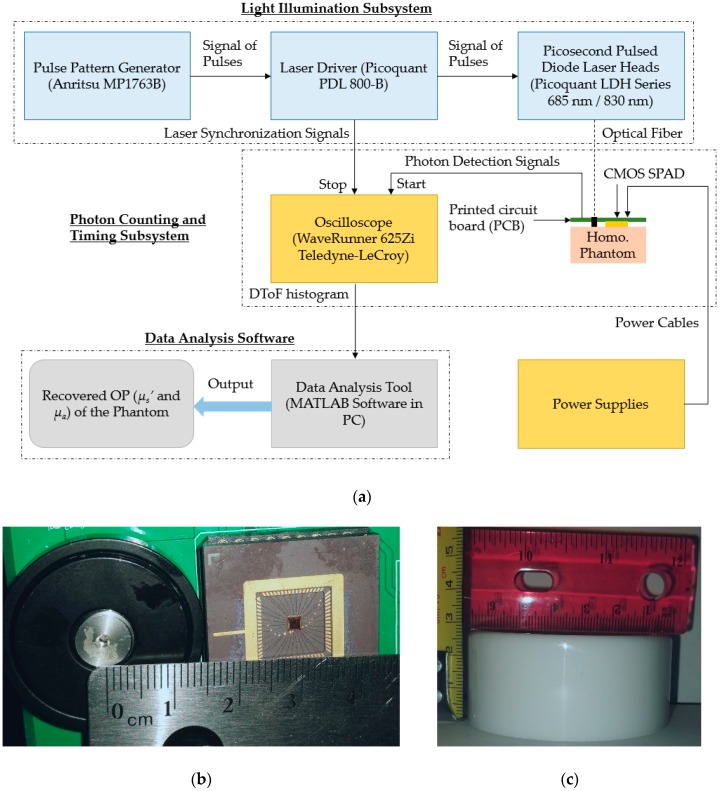
(**a**) Diagram of main components of the TR-DOS prototype; (**b**) light source and detector arrangement (28 mm source-detector distance) that are attached to the surface of phantoms; (**c**) a sample of the homogeneous phantoms.

Two picosecond pulsed diode laser sources are used to illuminate the phantoms at two wavelengths—685 and 830 nm—within the biological window [28]. These two chosen wavelengths of 685 and 830 nm are suitable to observe the concentrations for deoxy-hemoglobin (Hb) and oxyhemoglobin (HbO_2_) in tissues, respectively [5]. The maximum average optical power of the laser sources is ~10 mW [28]. A multi-mode optical fiber (four-meters long) is used to transmit light from each laser source to a phantom Figure 2a [29]. An external pulse generator (MP1763B, Anritsu, Atsugi, Japan) is connected to the laser driver to trigger the laser sources with a repetition rate (RR) that can vary from 1 Hz to 80 MHz [30,31]. Increasing the intensity and the repetition rate (RR, maximum is 80 MHz) for the laser driver increases the optical power of the light from the laser sources. The maximum average optical power of laser sources decreases by 5% to 15% due to the light losses through long optical fibers. Here, we used 50 MHz of RR of the pulses, that is, one laser pulse every 20 ns. This chosen RR allows the maximum average optical power of the illumination source to reach up to ≈ 6 mW if the highest intensity is used. This high RR increases the probability for detecting the re-emitted photons relative to the noise, thus improving the signal-to-noise ratio (SNR) of the measured DToF histograms.

### 2.2. Photon Counting and Timing Subsystem

The re-emitted photons from a phantom are collected by our custom designed FR CMOS SPAD detectors (100 μm^2^ active area) in reflectance geometry [25]. In the excess voltages used (V_ex_ = 1.2 V), our detector has short timing jitter (<150 ps), low dark count rate (DCR) ≈ 13 kHz at room temperature, and a photon detection efficiency (PDE) ≈ 0.6% at 685 nm and ≈ 0.2% at 830 nm [26]. Also, the dead-time of this detector is approximately 1 μs which allows for a maximum ≈ 0.5 million counts per second. The source to detector distance (SDD) between the fiber of the light source and the detector fixed at 28 mm, as shown in Figure 2b. The output signals of the FR CMOS SPAD and laser driver are connected to a Teledyne LeCroy oscilloscope (WaveRunner 625Zi, Teledyne LeCroy, Chestnut Ridge, NY, USA) to determine the delay between the edges of the two signals and record the photon time of arrival (PTA) for each detected photon [32]. Then, a DToF histogram can be performed by counting a reasonable number (~10^5^) of re-emitted photons [6].

### 2.3. Data Analysis Tool

**Figure 3:**
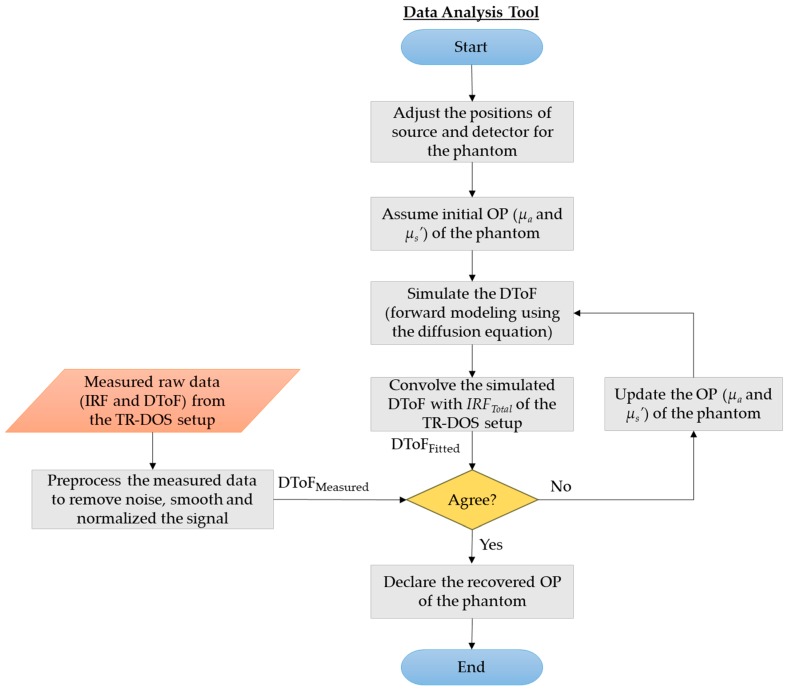
Flowchart of the data analysis process to recover the OP of the phantoms using the TR-DOS prototype.

We have developed an iterative inverse problem solver to preprocess the raw data (DToF histograms) and retrieve the OP of the measured phantoms based on MCXLAB capabilities [33]. This inverse problem solver uses an analytical solution of the diffusion equation (DE) for a semi-infinite medium to simulate the light propagation (the forward problem) and generate DToF histogram for each assumed *μ_a_* and *μ_s_*’ of a phantom. Before analyzing the raw data using this best fitting model, three steps of preprocessing are performed in sequence—noise removal from the signal, smoothing, and normalizing the DToF curve. In each iteration, the simulated DToF histogram is convolved with the *IRF_Total_* of the TR-DOS setup to give a fitted DToF histogram. Then, the fitted DToF histogram (already convolved with *IRF_Total_*) is matched to the experimentally measured DToF using a nonlinear least square solver (the Levenberg-Marquardt method). The iterative process starts with adjusted initial values of *μ_s_*’ and *μ_a_* that are typical of human tissues: *μ_s_*’ = 1.0 mm^−1^ and *μ_a_* = 0.01 mm^−1^ [34,35]. Other OP parameters such as the anisotropy factor (g = 0.9) and the refractive index (n = 1.5) are kept constant while the iterative process is running. Then, the iterative process continues until the inverse problem solver finds the best solution of the objective function. Figure 3 illustrates the steps of preprocessing and analyzing the raw data from the TR-DOS setup.

## 3. Characterization Methods

We evaluated the performance of our prototype using assessments based on two well-known protocols. The first protocol is the basic instrumental performance (BIP) that focuses on the characterization of the TR-DOS setup and its equipment without considering a measuring object (such as a phantom) [36]. The second protocol is MEDPHOT which evaluates the capability of TR-DOS prototype to recover the OP for homogeneous phantoms [37]. In this section, we describe the concepts and the experiments to characterize our TR-DOS prototype.

### 3.1. Basic Instrumental Performance Protocol

Following the BIP protocol, three parameters of the TR-DOS setup are measured. These parameters are the average delivered optical power on the phantoms (*P_Source_*) from the laser source, the differential non-linearity (DNL), and the total Instrument Response Function of the setup (*IRF_Total_*) [36].

#### 3.1.1. Light Power

An optical power meter (Model 1830-C, Newport, Irvine, CA, USA) was used to measure the power of the light from the fiber that transmits light from laser sources to phantoms [38,39]. Measurements were taken for both light sources with the same repetition rate (50 MHz) that were used to illuminate the phantoms. The illuminated areas (*A_source_*) on the surface of the phantoms were ≈ 3 mm^2^ for both light sources. The optical powers of the delivered light to the phantoms were 2.2 mW and 3.6 mW for the 685 nm and 830 nm laser sources, respectively. These levels of optical power are much lower than the maximum permissible exposure (MPE) for human skin. The MPE levels for skin are estimated as 6 mW/*A_source_* (3 mm^2^) for 685 nm and 11 mW/*A_source_* (3 mm^2^) for 830 nm laser beams. At each wavelength, we calculated the MPE according to the data acquisition time in the experiments (20 min for each phantom) using formulas reported in the literature [40].

#### 3.1.2. Differential non-linearity (DNL) of Photon Timing

**Figure 4:**
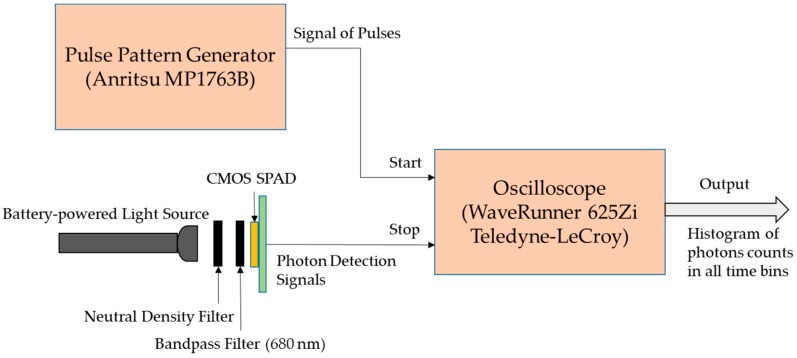
DNL measurement setup to acquire histograms of the PTA for all detected photons.

The differential non-linearity (DNL) is mainly used to estimate the non-uniformity of the width of the time bins in the photon timing equipment such as time-to-digital converter (TDC) or time-correlated single photon counting (TCSPC) [36]. The DNL is a routine test that is required for TR-DOS systems even if the photon time of arrival (PTA) is measured using a different equipment such as an oscilloscope. Ideally, the counted photons in each bin in the histogram should be equal [36]. However, during the experiments, there are differences in the distribution of the counted number of photons as a result of the DNL. We measured the DNL using a pulse pattern generator to send repetitive signals (50 MHz) as the start signal to the oscilloscope, and the FR CMOS SPAD to count photons and send the stop signal to the oscilloscope. It is worth noting that to measure the DNL, a battery-powered light source to illuminate the detector to prevent any electrical power-line interference is recommended [36]. Also, we placed neutral-density (ND) filters between the light source and CMOS detector to attenuate (~95%) the light and prevent saturation of the detector. An optical bandpass filter (680 nm) was used to allow only light at the required wavelength to reach the detector. Figure 4 illustrates the experimental setup for DNL measurements. It is recommended to measure PTA for more than 10^5^ counted photons in each time bin to obtain a good SNR and a more accurate estimation of the *ε_DNL_* using the following equation [36]:

(1) $$\varepsilon_{DNL}=\frac{N_{DNL,max}\left(t\right)-N_{DNL,min}\left(t\right)}{\bar{N_{DNL}}}$$

where *N_DNL,max_* and *N_DNL,min_* are the maximum and the minimum number of recorded photons in the time bins (maximum peak to minimum peak). $\bar{N_{DNL}}$ is the average number of counted photons in time bins.

#### 3.1.3. Total IRF of the TR-DOS Setup

The *IRF_Total_* is an important performance measure for TR-DOS systems, and the full-width-at-half-maximum (FWHM) of the *IRF_Total_* should be as short as possible, especially if short source-detector separations are used. Moreover, the FWHM of *IRF_Total_* must be less than 1.0 ns so as not to distort the raw data (DToF histograms) [41,42]. The FWHM of *IRF_Total_* represents the root of the sum of squared *IRF* for each instrument such as laser source, photon detector (timing jitter of the CMOS SPAD), optical fiber and photon timing equipment (WaveRunner 625Zi, Teledyne LeCroy, Chestnut Ridge, NY, USA) in our TR-DOS setup. The FWHM of *IRF_Total_* of this TR-DOS setup is given by [1]:

(2) $$\mathrm{FWHM}\;\mathrm{of}\;IRF_{Total}\approx \sqrt{IRF_{laser\;source}^{2}+IRF_{OpFb}^{2}+IRF_{CMOS\;SPAD}^{2}+IRF_{\mathrm{Oscilloscope}}^{2}}.$$

To measure the FWHM of *IRF_Total_* of the TR-DOS setup, the optical fiber connected to the laser source is placed in front of the CMOS SPAD detector, and a thin diffuser such as a sheet of white paper was used between the fiber and the detector to ensure scattering of the light [36,42,43]. Multiple scattering interactions happen for photons in the thin diffuser that vary the directions of detected photons with a negligible broadening of the measured FWHM of *IRF_Total_* [36].

### 3.2. Optical Properties Quantification of Homogeneous Phantoms

Following the MEDPHOT protocol, we used two assessments such as accuracy and linearity to characterize the OP for several homogeneous phantoms using the TR-DOS prototype. The measured DToF histograms (raw data) were preprocessed to remove the noise and smooth the DToF curves. After that, the measured DToF histogram for each phantom was analyzed using the best fitting model to estimate the OP of the phantom as described earlier in Section 2.3.

#### 3.2.1. Accuracy Assessment

The accuracy of the OP quantification is determined by comparing the true OP of the phantoms with the recovered OP. The accuracy of the retrieved *μ_s_*’ and *μ_a_* is estimated separately for each phantom by calculating the error using the following equation [37]:

(3) $$\varepsilon =\frac{OP_{recovered}-OP_{true}}{OP_{true}}.$$

The result from Equation (3) *ε* is converted to a percentage, determines the discrepancy in accuracy assessments.

#### 3.2.2. Linearity Assessment

The linearity test focuses on the changed values of the retrieved *μ_s_*’ or *μ_a_* when the true *μ_s_*’ or *μ_a_* are varied. The main sources of inaccuracies in the retrieved *μ_s_*’ or *μ_a_* are from three factors [44]. First, there are some small fluctuations of the time origin (*t*_0_) from one measurement to another, and these fluctuations impact on the accurate estimation of the OP, particularly *μ_s_*’. Second, there is a systematic distortion of the measured DToF histograms due to the impact of the *IRF* and the noise (background and false triggering). Third, there is some error in the theoretical approximations when the diffusion equation (DE), which is less accurate than the radiative transfer equation (RTE), is used simulate the light propagation in diffusive media [44,45].

#### 3.2.3. Preparation of Phantoms

To perform accuracy and linearity assessments, sets of nine homogeneous solid cylindrical phantoms were prepared for the measurements. The phantoms have three *μ_a_* (0.005 mm^−1^, 0.009 mm^−1^ and 0.013 mm^−1^) and three *μ_s_*’ (0.4 mm^−1^, 0.8 mm^−1^ and 1.2 mm^−1^) values. These phantoms are named according to their OP using letters for variable *μ_s_*’ (A, B, and C) and numbers for variable *μ_a_* (1, 2, and 3). Thus, the OP for phantom A1 are almost 0.005 mm^−1^ and 0.4 mm^−1^, and the OP for phantom C3 are almost 0.013 mm^−1^ and 1.2 mm^−1^ for *μ_a_* and *μ_s_*’, respectively. The range of OP for these phantoms has been chosen to be in the range of the known OP of human tissue [46]. The height of each phantom is 27 mm, and the diameter is 67 mm, as shown in Figure 2c.

Phantoms were prepared using epoxy-resin, titanium dioxide (TiO_2_), and India ink for the phantom matrix media, scattering agent and absorbing agent, respectively [47]. The concentrations of TiO_2_ and ink were varied linearly to produce changes of about 0.005 mm^−1^ and 0.4 mm^−1^ (at the wavelengths range between 685 nm and 830 nm) for *μ_a_* and *μ_s_*’, respectively. The TiO_2_ was suspended in an ethanol solution (ratio is 1 gm TiO_2_/ 3 ml ethanol) to ensure good mixing with the resin. To determine the required TiO_2_ and ink concentrations to produce specific OP of phantoms, we used a steady-state spatially resolved diffuse reflectance system with a custom-made inverse problem solver that was described in our previous work [10]. After fabricating the phantoms, the surfaces were polished using several sandpapers (grits vary from 120 to 600) to remove scratches. Then, we estimated the actual OP for each phantom using our time-resolved diffuse optical tomography system [10]. Table 1 summarizes the actual OP of the used phantoms in this work at the used two wavelengths 685nm and 830 nm.

#### 3.2.4. Data Acquisition and Preprocessing

**Figure 5:**
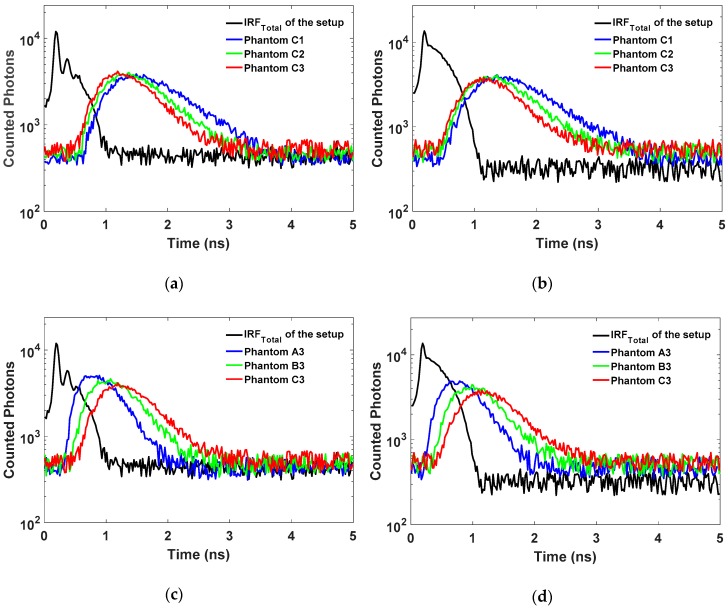
The *IRF_Total_* versus the measured DToF histograms for the phantoms: (**a**) high scattering phantoms with variable *μ_a_* at 685 nm; (**b**) high scattering phantoms with variable *μ_a_* at 830 nm; (**c**) high absorption phantoms with variable *μ_s_*’ at 685 nm; and (**d**) high absorption phantoms with variable *μ_s_*’ at 830 nm.

To measure the DToF histogram for each phantom, laser light is injected to the phantom, and the re-emitted photons are detected in reflectance geometry using an identical source to detector distance (SDD) of 28 mm. Each measurement is done for 20 min until the DToF histogram was acquired and around 600 K photons and background noise signal were counted. It is worth noting that this long time for data acquisition is not necessary since 200 s of data acquisition time is enough to count ≈ 10^5^ events (photons and noise). In our experiments, this slow photons timing process is a result of the limited update rate of the oscilloscope for the time base (20 ns) used. This slow update rate allows only for recording a small portion of the counted photons and noise from the SPAD detectors (~500 counts per second), whereas the maximum count rate of our SPAD detectors is up to ~0.5 million counts per second. Therefore, using a longer data acquisition time (20 min) is useful to increase the number of the counted photons to acquire smoother DToF curves to more accurately retrieve the OP. On the other hand, to use this prototype in real-time applications, the SPAD detectors should be connected to TDCs or a TCSPC module to acquire a raw data histogram and count ≥ 10^6^ photons within a few seconds. In this work, all the acquired DToF histograms have 20 ns range, and 1000 time bins (width of each bin is 20 ps). These measurements were taken three times (at different positions on the surface for each phantom) using two laser sources at 685 and 830 nm. Figure 5 shows the DToF histograms that were measured for high scattering phantoms and high absorption phantoms at 685 nm and 830 nm, and the corresponding *IRF_Total_*. In these figures, the variation of the dynamic range between the *IRF_Total_* and the measured DToF curves can be observed. The DCR (~13 kHz) of the detector versus the maximum count rate (500 K) restricts the dynamic range of this prototype to be 1.7 orders of magnitude for high-intensity light in the *IRF_Total_* measurements. This maximum level of the DR is lower than reported dynamic range for FR-TR-DOS systems by an order of magnitude [21]. The limited DR for our TR-DOS systems is a result of a relatively high percentage DCR (~2.5% of the maximum count rate) and the modest PDE of the SPAD detectors used. Therefore, it is noticed that the DToF curves have a lower order of magnitude of the DR due to the lower intensity of the measured light in DToF measurements.

**Figure 6:**
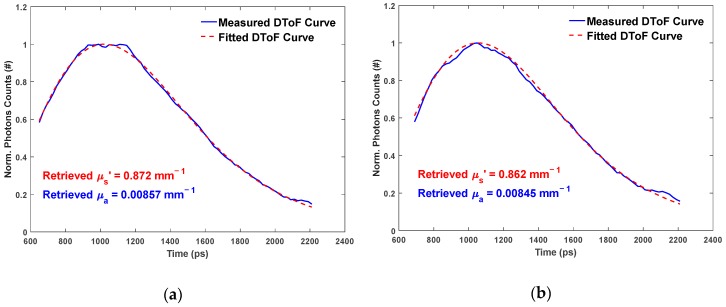
DToF_Measured_ curves and DToF_Fitted_ curves for B2 phantom with SDD = 2.8 cm: (**a**) at 685 nm; and (**b**) at 830 nm.

To prepare the DToF histograms for analysis, noise such as DNL distortion is removed from the signals. After that, each DToF curve is smoothed using a moving average filter for a span of seven time bins (representing 140 ps). The last step of preprocessing is the normalization for each DToF before starting the best fitting model process, as illustrated earlier in Figure 3. We compared the results (for the OP quantification) for different ranges of points in the DToF histograms that are involved in the best fitting process. Then, we chose the range of points from 60% of the DToF curve peak in the rising edge and 15% of the DToF curve peak in the falling tail because the most accurate OP quantification results were obtained using this range. Figure 6 shows an example of the fitting process for the same phantom (B2) at both wavelengths used. The time in the x-axis represents the delay between the DToF curve and the rising edge of the *IRF_Total_* (0 ps).

## 4. Results and Discussions

Several measurements were taken to characterize the TR-DOS prototype following the BIP and MEDPHOT protocols. In this section, we report and discuss the results of the following assessments: DNL, *IRF_Total_*, accuracy, and linearity. Then, we evaluate this prototype and describe its limitations.

### 4.1. Differential Non-Linearity

**Figure 7:**
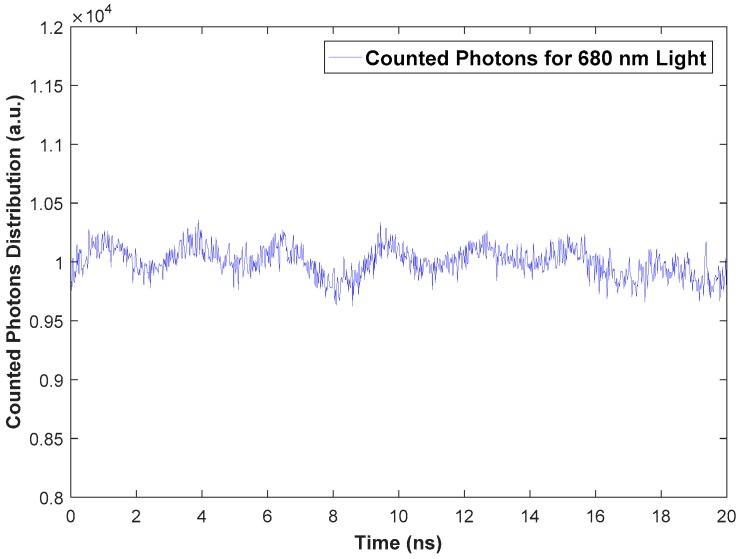
Histogram of PTA distributions for 10^7^ counted photons in all time bins (1000 bins for 20 ns range).

In the DNL measurements, 10^7^ photons were counted for all time bins (1000 bins and width for each bin is 20 ps). In Figure 7, the histogram of PTA distributions that was used to calculate the DNL is shown.

The ε_DNL_ was estimated to be 0.073 (7.3%) using Equation (1) and the measurement setup illustrated in Figure 4. This level of error is acceptable because the level of DNL error is normally several percents [36]. This result indicates that the oscilloscope performs time-to-digital conversion (for the counted photons) with a slight distortion of the width for time bins.

### 4.2. Total Instrument Response Function (IRF_Total_)

**Figure 8:**
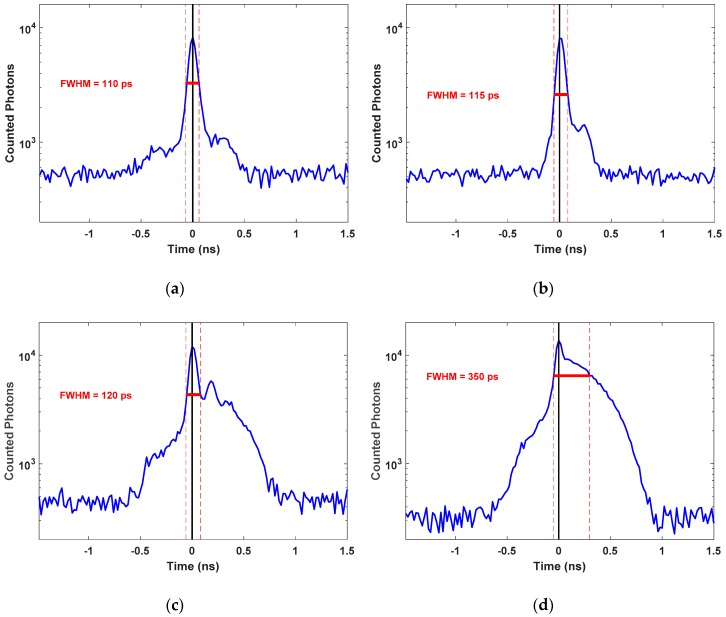
FWHM of *IRF_Total_* of the TR-DOS prototype at two different wavelengths used in this work: (**a**) 685 nm at low optical power (0.05 mW); (**b**) 830 nm at low optical power (0.06 mW); (**c**) 685 nm at the used optical power (2.2 mW) to illuminate the phantoms; and (**d**) 830 nm at the used optical power (3.6 mW) to illuminate the phantoms.

The narrowest FWHM of *IRF_Total_* in each wavelength is less than 120 ps for low-power light (Figure 8a,b). However, we used a higher power to illuminate the phantoms, which is essential to eliminate the impact of the CMOS SPAD’s modest PDE and the small active area. At a higher power level, the width of pulses for the picosecond diode laser is increased significantly, which leads to broadening of the FWHM of *IRF_Total_* of the TR-DOS setup [1,28]. Therefore, the temporal widths of *IRF_Total_* are broadened to 120 ps and 350 ps using 685 nm and 830 nm laser sources, respectively. Figure 8 shows the *IRF_Total_* that were measured at both wavelengths of 685 nm and 830 nm.

An accurate determination of the FWHM of *IRF_Total_* is essential because it will be convolved with the simulated DToF (from the forward modeling) to perform the fitted DToF (*DToF_Fitted_*) as follows [44,48,49].

(4) $$DToF_{Fitted}=DToF_{Simulated}*\mathrm{FWHM}\;\mathrm{of}\;IRF_{Total}$$

### 4.3. The Accuracy of the OP Quantification

In the accuracy assessment, we estimated the percentage of error for the recovered OP against the actual OP for each phantom. Table 2 shows the results of accuracy assessments for all nine phantoms at the two wavelengths.

The percentage of errors were estimated using Equation (3), and the average of errors were 6.5% and 9.5% for *μ_s_*’ and *μ_a_*, respectively. These levels of error in OP quantification is a known limitation for DOS techniques [44,45,48]. The highest discrepancy for *μ_s_*’ appeared in the phantom (A3) at 685 nm and phantom (A2) at 830 nm that reached up to 12% and 19%, respectively. The main reason for these errors comes from the difficulty to discriminate changes in the shapes of the DToF curves for low *μ_s_*’ turbid media [45]. Also, the percentage errors for the retrieved *μ_s_*’ have increased for low scattering phantoms (A) due to the low values of *μ_s_*’, as shown in Table 2. On the other hand, the average discrepancy for retrieved *μ_a_* decreases significantly at 685 nm in high *μ_s_*’ phantoms (C). Overall, the estimated accuracy for homogeneous phantoms using this prototype is similar to the reported levels of accuracy of TR-DOS measurements for prototypes that use commercial detectors [37,49,50].

### 4.4. The Linearity of the OP Quantification

**Figure 9:**
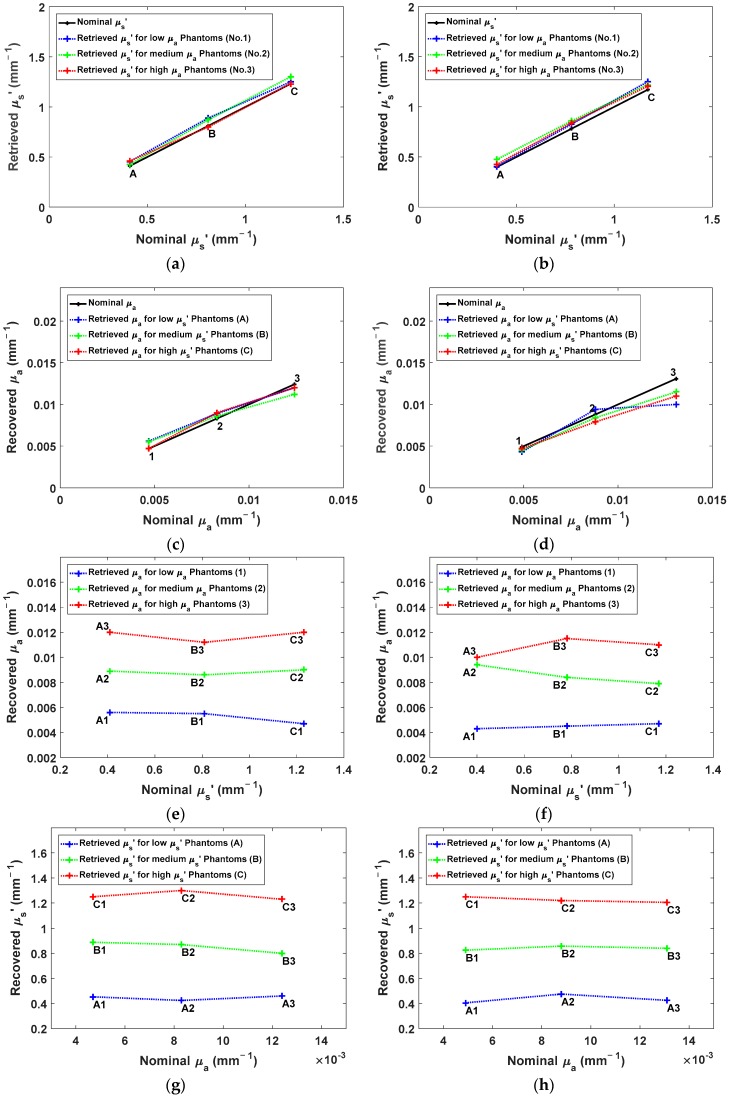
The linearity of the retrieved OP versus the actual OP for nine phantoms at two different wavelengths: (**a**) and (**b**) represent the retrieved *μ_s_*’ against the actual *μ_s_*’ at 685 and 830 nm, respectively; (**c**) and (**d**) represent the retrieved *μ_a_* against the actual *μ_a_* at 685 and 830 nm, respectively; (**e**) and (**f**) represent the retrieved *μ_a_* against the actual *μ_s_*’ at 685 and 830 nm, respectively; and (**g**) and (**h**) represent the retrieved *μ_s_*’ against the actual *μ_a_* at 685 nm and 830 nm, respectively.

The TR-DOS prototype has presented good linearity for retrieving *μ_s_*’ with some overestimation for all nine phantoms at both wavelengths Figure 9a,b. In addition, the prototype has shown good linearity for retrieving *μ_a_* for phantoms B and C in both wavelengths with some underestimation at 830 nm Figure 9c,d. On the other hand, better linearity was obtained for the retrieved *μ_a_* for low *μ_a_* phantoms (1) versus high *μ_a_* phantoms (2 and 3). This is a result of the reduction in the absorption-to-scattering coupling at both wavelengths Figure 9e,f. Also, the results show good linearity for the retrieved *μ_s_*’ for all phantoms which indicates low scattering-to-absorption coupling (Figure 9g,h). Generally, the linearity of this TR-DOS prototype is very good and comparable to the reported linearity results in the literature [37,49,50,51,52].

### 4.5. Evaluation of this Prototype and the Potential Applications 

The evaluation of this TR-DOS prototype has demonstrated good performance, and it can be used for tissue optics applications for the following reasons. First, the chosen optical power of the illuminated light (much lower than MPE of skin) were useful to overcome the modest PDE and the small active area of the detector by increasing the number of injected photons and accordingly increasing the number of detected photons. Second, the low errors for DNL and the narrow *IRF_Total_* verified that raw data (DToF histograms) could be generated with good accuracy (without distortion) for human tissues such as muscle, breast or a newborn’s head. However, the long time for data acquisition and the modest dynamic range (one order of magnitude) for DToF curves, as shown in Figure 5, are limitations in the use of this prototype for some tissue optics applications such as functional brain imaging. Therefore, it is necessary to develop high temporal resolution TDC and integrate it with this FR CMOS SPAD detector to reduce the data acquisition time to the range of few seconds. This will enable this prototype to observe physiological changes (such as blood oxygen saturation) in tissue which happen within seconds. Fast data acquisition time is not expected to improve the dynamic range significantly due to the low PDE, and small active area for the detector used. Therefore, this prototype will probably not be capable of observing the change of the OP in the deep regions in multilayered tissue such as gray matter and white matter in an adult’s head. Here, the OP mainly depend on the late photons in DToF curves and require a fast time gating detection capability. Third, the results of accuracy and linearity assessments indicated that this prototype could be used to distinguish between pathological tissue and healthy tissue due to the noticeable OP variations between them. Overall, there are some potential improvements that can be achieved to design better FR SPAD detectors in 130 nm CMOS for TR-DOS applications.

### 4.6. Potential Developments of SPAD Detectors for Tissue Optics Applications

The SPAD detectors used here have demonstrated good performance in TR-DOS measurements. However, there are some issues that should be considered when SPAD detectors are being designed (particularly in 130 nm CMOS) to improve their performance and overcome some limitations for TR-DOS. The important FR-CMOS SPAD features in TR-DOS measurements are the following: First, the most important features for each designed pixel are short timing jitter and low noise sources such as the DCR and after-pulsing. Increasing the size of the active area and the thickness of the depletion region increases the PDE of the detector. However, the size of the active area for each pixel and the thickness of the depletion region should not be enlarged too much to avoid increasing the timing jitter and noise. For instance, in this work, short timing jitter of the detector used enables the TR-DOS system to achieve short *IRF_Total_* that was sensitive to the width and the shape of the pulses of the laser sources at different optical powers. Also, short *IRF_Total_* is important to achieve good accuracy for retrieving the OP in TR-DOS applications. Second, reducing the dead time is desirable if we want to increase the count rate for each CMOS pixel and avoid saturation of the detector when many photons are impinging on the active area during a relatively long dead time. However, the typical dead time for CMOS SPAD pixels (tens of ns up to few *μs*) is enough to count more than 10^5^ photons/s. This count rate of photons is compatible with TCSPC modules and TDCs units that suffer from pile-up effect if the count rate of photons exceeds 5% of the RR of the reference signal such as the laser repetition rate in TR-DOS systems [53]. Third, the impact of a modest PDE of the CMOS SPAD pixels can be eliminated by increasing the optical power of the illuminated light and using a small SDD (1 cm–3 cm). Fourth, using an array of SPAD pixels is vital to enlarge the total active area of the SPAD detectors and increase the number of counted photons in TR-DOS measurements, especially when a larger SDD (> 3.0 cm) is used. However, a large number of an array of SPAD pixels is not necessary to increase the number of the counted photons since each TDC will be shared by several pixels to avoid reducing the fill factor of the pixels. Sharing TDCs by a large number of pixels will ultimately lead to an increase in the data acquisition time and keep a limited number of the array’s pixels active during the measurements. Therefore, a CMOS SPAD detector with a reasonable number of pixels (~100) such as in a 1 D array or a 2 D array (10 × 10), where each pixel has an independent TDC, can be useful for TR-DOS measurements using illuminated light with optical power lower than the MPE of the skin. For a TR-DOS, a 1 D line array of pixels is preferable to keep the pixel electronics outside the pixel to increase the fill factor of the SPAD detectors [20]. Fifth, to maintain a good level of SNR, SDD should be reasonable (≤ 4 cm) in TR-DOS measurements without exceeding the MPE of the light for the skin. Otherwise, the DR of the measured DToF curves will be significantly degraded, and the measured DToF curves will not be valid to recover the OP for the measured object. Sixth, another possible approach to improve the achievable DR of the TR-DOS prototype is to reduce the percentage of DCR versus the maximum count rate. Therefore, from our perspective, we believe that a TR-DOS prototype using FR CMOS SPAD detectors with DCR lower than 0.1% of the maximum count rate can acquire DToF with DR higher than two orders of magnitude. Such a TR-DOS prototype will achieve better depth sensitivity to recover the OP for deeper regions in tissues.

## 5. Conclusions

In the recent years, significant efforts were made to reduce the complexity, cost and size for time-resolved diffuse optical spectroscopy (TR-DOS) systems. Here, we described and characterized a TR-DOS prototype using low cost, compact, custom-designed free-running (FR) single-photon avalanche diode (SPAD) detectors in standard silicon 130 nm CMOS technology. This prototype was used to successfully perform distribution of time of flight (DToF) histograms in reflectance geometry for phantoms that have optical properties (OP) in the range of human tissues. The detector was used to acquire histograms using a low-power pulsed laser light with power levels below the maximum permissible exposure for human skin. The differential non-linearity was acceptable (7.3%) for photon timing with a temporal resolution in the range of tens of picoseconds, which is required for TR-DOS systems. The temporal widths of the total instrument response function of TR-DOS prototype were short enough to ensure that DToF histograms are not distorted and valid to be used to quantify the OP of homogeneous phantoms accurately. The results of the accuracy assessment for quantifications of the OP were very good for the realistic phantoms used, and the levels of error are within the range of results reported in the literature. The results of the linearity assessment demonstrate the potential of the prototype to observe the differences of the OP among several homogeneous phantoms. However, the long time for the data acquisition is a limitation of this TR-DOS prototype, but it can be shortened significantly by incorporating time-to-digital converters with the SPAD detectors on the same chip to perform the DToF histograms. Then, multichannel TR-DOS can be built using several low-cost photon-timing subsystems with FR silicon SPADs with TDCs in the same chip. Such a system would be very suitable for clinical applications such as functional newborn brain and muscle monitoring and optical mammography, particularly if the possible improvements of the SPAD detectors are used.

## Figures and Tables

**Table 1:** The nominal OP of the measured phantoms in this work.

Phantom	685 nm		830 nm		n
	*μ_s_*’ (mm^−1^)	*μ_a_* (mm^−1^)	*μ_s_*’ (mm^−1^)	*μ_a_* (mm^−1^)	
A1	0.41	0.0047	0.4	0.0049	1.5
A2	0.41	0.0083	0.4	0.0088	1.5
A3	0.41	0.0124	0.4	0.0131	1.5
B1	0.81	0.0047	0.78	0.0049	1.5
B2	0.81	0.0083	0.78	0.0088	1.5
B3	0.81	0.0124	0.78	0.0131	1.5
C1	1.23	0.0047	1.17	0.0049	1.5
C2	1.23	0.0083	1.17	0.0088	1.5
C3	1.23	0.0124	1.17	0.0131	1.5

**Table 2:** Relative errors in the accuracy assessment of the retrieved *μ_s_*’ and *μ_a_* versus the actual *μ_s_*’ and *μ_a_* for nine phantoms at two different wavelengths.

	% Errors in the Estimate of *μ_s_*’			% Errors in the Estimate of *μ_a_*		
**685 nm Results**	***μ_s_*’**					
*μ_a_*	**A (0.41)**	**B (0.81)**	**C (1.23)**	**A (0.41)**	**B (0.81)**	**C (1.23)**
1 (0.0047)	10.5	9.5	1.6	19	17	0
2 (0.0083)	3.5	7.5	5.7	7.2	3.6	8.4
3 (0.0124)	12	−1.2	0	−3.2	−9.7	−3.2
**830 nm Results**	***μ_s_*’**					
*μ_a_*	**A (0.4)**	**B (0.78)**	**C (1.17)**	**A (0.4)**	**B (0.78)**	**C (1.17)**
1 (0.0049)	1.3	5.8	6.8	−12.2	−8.2	−4
2 (0.0088)	18.8	10	4.3	6.8	−4.5	−10.2
3 (0.0131)	6.3	7.7	3	−23.7	−12.2	−16
